# Autism and the Case Against Job Interviews

**DOI:** 10.1007/s12152-024-09563-4

**Published:** 2024-05-13

**Authors:** Bouke de Vries

**Affiliations:** 1 Department of Philosophy, Ghent University, Ghent, Belgium; 2 Department of Philosophy, University of Zurich, Zurich, Switzerland

**Keywords:** Autism, Unemployment, Job interviews, Neurodiversity, Job simulation activities, IQ tests

## Abstract

Unemployment rates among autistic people are high even among those with low-support needs. While a variety of measures is needed to address this problem, this article defends one that has not been defended in detail and that has profound implications for contemporary hiring practices. Building on empirical research showing that job interviews are a major contributor to autistic unemployment, it argues that such interviews should be abolished in many cases for autistic and non-autistic people alike.

## Introduction

Autism is a common neurological condition^1^ that according to the fifth edition of the Diagnostic and Statistical Manual of mental disorders (DSM-5) is marked by ‘persistent deficits in social communication and social interaction across multiple contexts’ and by ‘restricted, repetitive patterns of behavior, interests, or activities’ [3]. Whereas some autistic people need little, if any, support from others to live autonomously, others require a lot, which explains why autism is frequently described as a ‘spectrum disorder’ or, more neutrally, as a ‘spectrum condition’. Still, even those with low-support-needs face important challenges, prominent among which is the subject matter of this article, namely that of *securing a job*.

Consider the situation within the United States. Notwithstanding the introduction of the American with Disabilities Act in 1990, a federal law whose aim is to prevent employment-related discrimination against persons with disabilities, some surveys report that only 58% of autistic Americans ever worked between high school and their early 20 s [4], which is not only less than the share of the general population who did but also than that of people with speech or language impairments (91%) and that of people with learning disabilities (95%). Similar patterns exist in other countries. For example, a mere 21.7% of autistic people in the United Kingdom were employed in 2020 compared to 81.3% of people without disabilities [5] – supposing for a moment that autism can be classified as a disability, which some have questioned (cf. [6]) – while in Australia, the labor force participation rate was 38% among the 94,600 people of working age (15–64 years) in 2018 compared to 53.4% of all working age people with disability and 84.1% of people without disability [7].

Although a variety of measures is needed to address autistic unemployment [8–11], the current article defends one that has not been defended in detail and that has profound implications for contemporary hiring practices. Drawing on empirical research showing that job interviews are a major contributor to autistic unemployment, it argues that such interviews should be abolished in many cases. To vindicate this claim, Section "Reasons for Putting Job Interviews to Scrutiny" offers evidence that interview-requirements are hampering the chances of many autistic individuals to secure employment, thus creating a presumption against their use. Section "The Case Against Job Interviews" suggests that it is very difficult to overcome this presumption. The reason is that more autism-friendly selection tools are generally available, namely cognitive tests and/or job simulation tasks, that (i) appear to be at least as effective and efficient, and (ii) to which job interviews are unlikely to add much value. Section "Abolishing Job Interviews vs. Granting Exemptions" goes on to explain why the use of these alternative tools ought to be preferred to exempting autistic applicants from job interviews, before concluding the article in Section "Final Remarks".

## Reasons for Putting Job Interviews to Scrutiny

Why is unemployment so high among autistic populations? Empirical scholarship on this topic reveals that one of the principal causes is that a large portion of autistic individuals struggles to make a good impression at job interviews [11–14], which is true even among those who participated in employment-readiness programs with interview-trainings – for example, one study from the US reports that in 2014, only 60% of 18,000 autistic individuals enrolled in such programs managed to land themselves a job [15]. These difficulties have been attributed to the fact that the interview is a part of the recruitment process during which applicants are expected to do things, or to have done things prior to the interview, that autistic people tend to find (particularly) challenging [11, 13]. Without attempting to provide an exhaustive list, one might think of practices such as grooming,shaking hands; understanding facial expressions; using expressive language; listening without interrupting; making eye contact; considering ‘what if’ scenarios; answering abstract self-reflective questions; finding the appropriate level of formality; and judging how much information to give when questions are open ([9, 11, 16, 17], pp. 4210–4211). Besides the difficulties posed by these activities, an exploratory study on the experiences of autistic adults in the American workplace (*N* = 87) found that a significant share of participants (16%) suffered from interview anxiety [13], which might further hinder performance and deter some autistic individuals from seeking employment altogether.

What is pertinent for us is that if job interviews are indeed a major contributor to autistic unemployment, as I assume from hereon, then it becomes imperative to ask *whether such interviews can be morally justified*. (An important related question here is whether ask whether they can be *legally justified* in countries with disparate impact laws such as the UK and US^2^; for the purposes of this contribution, my focus will be on their moral credentials.) Part of the reason for this is that being employed tends to provide us with highly valuable goods. Whereas some of these goods are instrumental, such as wages and employer-based health insurance, others are non-instrumental, such as improved wellbeing [18–20], professional identities and routines that can give meaning and structure to one’s life [21, 22], and social interaction with fellow workers [23, 24]. Another part is that being unemployed often has a negative impact on *other stakeholders*. Among these are people’s spouses and children, whose wellbeing and socio-economic situation is in many cases adversely affected [25, 26]. However, they also include members of the wider society [27], who might not only be required to help fund unemployment benefits but also miss out on the economic contributions a portion of the unemployed could have made, whereby it is worth noting that certain common autism-related traits, such as trustworthiness,integrity; attention to detail; and low absenteeism, enable a significant portion of autistic individuals to be especially productive in certain professions, such as those of data scientists, forensic accountants, laboratory technicians, computer programmers, equipment engineers, assembly-line workers, mail processors, librarians, automobile mechanics, package handlers, and archivists [14, 28–31].^3^

## The Case Against Job Interviews

What, if anything, can justify subjecting autistic people to job interviews in light of the micro-level and macro-level costs just mentioned? The most, and it would seem only, plausible justification if we accept basic meritocratic principles (cf. [32]) is that such interviews are a *proportionate means (i.e. one whose benefits outweigh its costs) for identifying the best qualified candidat*e. Assuming this to be correct, the remainder of this contribution argues that their use often is not proportionate.

In order to bring this out, we should begin by noting that when job interviews are unstructured, i.e. when applicants are not asked (mostly) the same questions and/or where there is not a fixed standard for assessing responses, it is doubtful whether they help to find the best qualified candidate. This is because there exists by now a large body of research indicating that such interviews are a poor tool for ascertaining and comparing people’s job qualifications [33–37],^4^ especially – but not exclusively – for those of autistic individuals [9]. But that is not all; even in cases where, structured or unstructured, job interviews are effective to a certain degree, there remain grounds for thinking that they frequently fail the proportionality-test due to the availability of more autism-friendly alternatives that are not any less effective or more expensive.

One such alternative involves the use of job simulations, which are standardized tasks (i.e. tasks administered and scored in a uniform manner; [39], p. 535) that mimic work deemed essential to the advertised jobs. For example, depending on the job on offer, applicants might be asked to respond to phone calls; handle grievances within a set amount of time; replenish shelves; take someone’s (fake) order; perform in a sales pitch; cut someone’s hair; do a typing exercise; repair an electronic device; give a university or school lecture; design a website; write code; or complete an onsite construction task. To see that such tasks are more accommodating of autistic applicants than are job interviews, it should be noted that they tend to place less weight on social skills, such as making eye contact and reading facial expressions, and do not normally require applicants to answer self-reflective questions.^5^ In terms of their effectiveness, a review by Schmidt and Hunter [37] spanning 85 years of research on personnel selection concluded that job simulations *surpass* job interviews in predicting job performance. Specifically, the study showed job simulations as having a correlation coefficient of 0.54 compared to 0.51 for job interviews. Finally, as far as efficiency is concerned, such simulations do not seem to fare worse either. The 30 min to 1.5 h that most job interviews last normally provide enough time for applicants to perform informative simulative tasks, including those mentioned above. And while designing and assessing job simulations can be laborious if done properly, these workloads are not greater in any obvious sense than those of constructing and assessing job interviews insofar as such interviews are structured (as they should be given the aforediscussed ineffectiveness of unstructured interviews).

It might be replied, correctly, that job simulations are of little use when applicants do not (yet) possess the skills and/or knowledge necessary for performing tasks related to the vacancy at hand (cf. [37, 40]). Since job interviews do not require such specialized skills or knowledge from applicants, a critic may infer that, in these cases, (structured) interviews offer the only viable selection method beyond resume screening.

The reason this is too quick is that *even under the conditions described*, an alternative method will usually be available that not only seems viable but *preferable* to conducting job interviews, namely the use of General Mental Abilities (GMA) tests. Such intelligence tests, which at least in the case of the increasingly popular computer-based gamified versions have been found to offer a ‘fair means for evaluating autistic candidates’ [41], p. 8), are more autism-friendly than job interviews by virtue of the fact that they do not expect autistic applicants to demonstrate their social skills. At the same time, they consistently emerge in personnel-studies as the single-best predictor of job performance [40, 42], p. 2 [37], and are comparatively cheap to administer, demanding little more than pen and paper or access to a computer (cf. [37], p. 264, [39], p. 538).

At this point, our critic might maintain that I am raising a false dilemma. Rather than being forced to choose between job interviews and the more autism-friendly screening tools discussed (i.e. job simulations and GMA tests), she might argue that employers could deploy these more autism-friendly selection methods *alongside* job interviews to maximize their chances of finding the best qualified candidate.

Two responses are in order. The first is that not every employer will have the resources (e.g. time, money, personnel) or the willingness to utilize both types of methods. When this is the case, my argument suggests that they should favour the identified interview-substitutes to avoid gratuitously burdening autistic people.

Yet, and this brings us to the second response, even when employers are able and willing to combine job interviews with more autism-friendly selection methods, the former’s use still needs to have *significant added value* for its costs to be proportional (see my earlier comments this section), which is something it appears to lack in many if not most cases. For one thing, Schmidt and Hunter’s review finds that GMA tests and work simulations combined are as predictive of work performance as the combination of GMA tests and structured job interviews – in both cases, the validity is 0.63 [37], p. 266). For another, the high validity of GMA tests (0.51) and the likely correlation between the predictive values of job simulations and job interviews suggest that, despite the lack of studies combining all three methods—GMA tests, job simulations, and job interviews—the incremental predictive value added by job interviews when used alongside GMA tests and job simulations can be expected to be small.

## Abolishing Job Interviews vs. Granting Exemptions

I have argued that the circumstances under which it is appropriate to have autistic people undergo job interviews are severely limited due to the availability of more autism-friendly selection methods to which such interviews are unlikely to add much value. However, this leaves open whether employers should not interview *anyone* in cases where interview-requirements cannot be morally justified towards autistic people or whether they may also simply exempt these individuals from such requirements. With Brian Barry [43], let us call this last strategy the ‘rule-and-exemption approach’.

There are contexts where exempting autistic individuals from rules looks morally permissible and possibly even necessary. For instance, while maintaining classroom order might require that students wishing to leave the classroom ask their teacher for permission to do so, exempting (hypersensitive) autistic students from this rule seems fitting, given that they may need to quickly retreat to a quiet place if and when their senses become overloaded [8]. The reason I nonetheless doubt whether the rule-and-exemption approach is justifiable within our context is twofold.

First, exempting autistic applicants from job interviews creates *epistemic asymmetries* that are likely to be disadvantageous to autistic people overall. This follows from the facts – but perhaps not exclusively from these facts – that (i) more information will be gathered about non-autistic candidates; (ii) employers generally seek to reduce the risk of hiring unqualified or mediocre candidates even if this is not their only objective; and (iii) the more information they possess about a given candidate, the easier it will be for them to tell whether that person is unqualified or mediocre.

Of course, insofar as poor performance at job interviews is an important contributor to autistic unemployment, as we saw it appears to be, exempting autistic applicants might still be preferable to *subjecting everyone to an interview*. Rather than challenging this, my point here is simply that granting such exemptions is unlikely to be desirable when *another option is on the table*, namely that of abolishing job interviews for everyone and possibly replacing them with job simulations or GMA-tests.

Second, the fact that the rule-and-exemption approach requires applicants to *disclose their autism* in order to receive an exemption raises several problems. One is that many autistic people do not want their (would-be prospective) employer to know that they are autistic. For example, a survey by Lindsay et al. [44] found that among four studies that included rates of workplace disclosure, between 25 and 69% of autistic employees had not disclosed their condition, which is often motivated by fears of stigmatization and discrimination [10, 45].^6^ Another problem is that such disclosures can, and sometimes do, have adverse consequences for how autistic people are treated on the work floor (suggesting that fears of stigmatization and discrimination are warranted in at least a subset of cases) [13, 45, 48].^7^ What is apposite for present purposes is that by abolishing job interviews for autistic and non-autistic job candidates alike, *both these problems are avoided.*^8^

## Final Remarks

After providing evidence that job interview-requirements are a significant contributor to autistic unemployment, one that this puts the onus of justification on their defenders, this article argued that it is doubtful whether there exist many situations where subjecting autistic applicants to these requirements can be justified. As became clear, this is due to the fact that more autism-friendly selection methods are usually available, namely job simulations, GMA-tests, that (i) do not seem to be any less effective or more expensive, and (ii) to which job interviews are unlikely to add much value. In addition to this, it was found that abolishing job interviews for autistic applicants and non-autistic applicants alike is preferable to offering exemptions from such interviews to the former. Apart from the fact that granting such exemptions create epistemic asymmetries between candidates, we saw that it requires autistic people seeking an exemption to disclose their condition, which was found to be problematic as well.

Let me end by noting that although these considerations suggest that many employers should refrain from using job interviews, it will be important for future research to consider whether replacing such interviews with more autism-friendly selection methods might have adverse impacts on other groups with high unemployment rates. My hope is that this article will inspire other scholars to work on this question.

## Data Availability

This paper has no associated data.

## Footnotes

1 Recent studies suggest that 2.5% of US children and adolescents are autistic [1] and that the total prevalence of autism in Asia might be as high as 3.9% [2].

2 See Section 19 of the UK’s Equality Act of 2010 and title VII of the US’s Civil Rights Act of 1964.

3 In line with this, one cost-benefit analysis has concluded that ‘enhancing the opportunities for adults with ASD to join the workforce is beneficial from […] a strict economic standpoint’ [27], p. 11).

4 But compare Chauhan [38].

5 The exception being cases where such skills are central to the positions on offer, as they might be when jobs for e.g. salespersons and food servers are advertised that involve intensive social interaction with neurotypical strangers.

6 In some cases, such fears cause autistic people to try to act as a neurotypical person, which is phenomenon known as ‘camouflaging’[46, 47].

7 In saying this, I am not denying that there can also be benefits to disclosing one’s autism to potential prospective employers. For example, some studies have found that such disclosures can improve judgements about the performance of autistic applicants in job interviews [49, 50].

8 Which is not to rule out that employers and/or governments should simultaneously address the anti-autistic prejudices that are the root of the disclosure-related problems just mentioned, for example by hosting workshops on neurodiversity and by teaching children about neurodivergent traits in school (cf. S. [51]). While abolishing job interviews is one important measure for tackling autistic unemployment, it is certainly not the only one that is due.
